# *HER2 *gene status in primary breast cancers and matched distant metastases

**DOI:** 10.1186/bcr1676

**Published:** 2007-05-19

**Authors:** Coya Tapia, Spasenija Savic, Urs Wagner, René Schönegg, Hedvika Novotny, Bruno Grilli, Michelle Herzog, Audrey DeVito Barascud, Inti Zlobec, Gieri Cathomas, Luigi Terracciano, Georg Feichter, Lukas Bubendorf

**Affiliations:** 1 University Department of Pathology of Basel and Baselland, Institute for Pathology, Schönbeinstrasse 40, Basel, 4003, Switzerland; 2 Viollier AG, Division of Histopathology and Cytology, Jacob Burckhardt-Strasse 86, Basel, 4002, Switzerland; 3 Cantonal Hospital, Institute for Pathology, Rorschacher Strasse 95, St Gallen, 9007, Switzerland; 4 University Department of Pathology of Basel and Baselland, Cantonal Institute for Pathology, Mühlemattstrasse 11, Liestal, 4410, Switzerland

## Abstract

**Introduction:**

The status of the gene encoding human EGF-like receptor 2 (*HER2*) is an important prognostic and predictive marker in breast cancer. Only breast cancers with *HER2 *amplification respond to the targeted therapy with trastuzumab. It is controversial to what degree the primary tumour is representative of distant metastases in terms of *HER2 *status. Discrepancies in *HER2 *status between primary tumours and distant metastases have been described, but their reasons remain unclear. Here, we compared *HER2 *status on cytological specimens of distant metastases with the result from the primary carcinomas, and explored the prevalence of and the reasons for discrepant results.

**Methods:**

*HER2 *status was determined by fluorescence *in situ *hybridisation. *HER2 *gene amplification was defined as a *HER2*/chromosome 17 signal ratio of 2 or more. *HER2 *results from cytological specimens of matched distant metastases were compared with the results from the corresponding primary tumours (*n *= 105 patients). In addition, lymph node metastases were analysed in 31 of these patients.

**Results:**

*HER2 *amplification was found in 20% of distant metastases. *HER2 *status was discordant between the primary tumour and distant metastasis in 7.6% of the 105 patients. Re-evaluation revealed that in five patients (4.7%), discrepancies were due to interpretational difficulties. In two of these patients, focal amplification had initially been overlooked as a result of heterogeneity in the primary tumours or in the metastases, respectively. A further three patients had borderline amplification with a ratio close to 2. Discrepancy remained unexplained in three patients (2.9%).

**Conclusion:**

*HER2 *gene status remains highly conserved as breast cancers metastasise. However, discrepant results do occur because of interpretational difficulties and heterogeneity of *HER2 *amplification. Cytological specimens from distant metastases are well suited for *HER2 *fluorescence *in situ *hybridisation analysis.

## Introduction

The *HER2 *oncogene encodes a transmembrane tyrosine kinase (human EGF-like receptor 2) located on chromosome 17q21 [1,2]. HER2 protein belongs to the epidermal growth factor family [3] and is important for cell differentiation, adhesion and motility [4]. *HER2 *gene amplification and protein overexpression exists in about 20% of breast cancers [5,6] and is linked to a poor prognosis [7]. From a clinical point of view, HER2 receptor has become important as a target for antibody-based therapy with trastuzumab (Herceptin^®^) [8]. Trastuzumab was originally approved for the treatment of metastatic *HER2*-positive breast cancers because of a significant survival benefit when given in combination with paclitaxel [9-11]. More recently, adjuvant treatment of primary, *HER2*-positive breast cancers with trastuzumab has been shown to improve patient outcome markedly [12]. Thus, determination of *HER2 *status in every breast cancer patient to select for adjuvant treatment with trastuzumab is becoming a standard worldwide. There has been much debate on how to test *HER2 *status. Other than fluorescence *in situ *hybridisation (FISH), immunohistochemistry is liable to technical and interpretational variability [13-15]. Accordingly, it has been shown that response to trastuzumab is strongly associated with *HER2 *gene status irrespective of protein expression determined by immunohistochemistry [16,17]. Importantly, no standardised immunocytochemical assay is available for cytological specimens. For these reasons, testing the *HER2 *gene status by FISH is now widely regarded as the gold standard [18].

*HER2 *status is commonly determined in the primary tumour, because biopsies from metastasised lesions are not always available. In three previous FISH studies on 12 to 68 patients, the prevalence of a discrepant result between the primary breast cancer and the distant metastasis ranged from 0% to 22.2% [19-23].

Because of the controversial data and the therapeutic importance of *HER2 *testing, we compared the *HER2 *status in a large series of primary breast cancers and their matched distant metastases by FISH. In addition, we attempted to elucidate reasons for these discrepancies.

Our results demonstrate that discrepancies in *HER2 *gene status between primary breast cancers and matched metastases do occur and may be related to technical and interpretational difficulties.

## Materials and methods

### Patients and specimens

A consecutive series of 105 cytological specimens from distant metastases of breast cancer samples was obtained from the institute for Pathology of the University Hospital Basel (*n *= 92), the Cantonal Institute for Pathology in Liestal (*n *= 7) and the Cantonal Institute for Pathology in St Gallen (*n *= 6), Switzerland. The specimens had been collected between the years 1999 and 2006. Data on pT (tumour size and invasion) and pN (lymph-node status) categories were obtained from the original pathology reports. Because systematic histological grading according to Bloom Richardson Elston [24] was not available in all patients, we used the nuclear grade (1 to 3) that was described in all cases. A tissue block containing representative tissue from each of the primary breast cancers was retrieved from the archives of the above-listed institutes as well as from Viollier AG, Division of Histopathology, Basel. A tissue microarray (TMA) was constructed from these original blocks as described previously [25]. The TMA included 87 primary breast cancers and 38 matched lymph node metastases that had not been previously analysed by FISH. One tissue core with a diameter of 0.6 mm was sampled from each of the primary tumours. Because lymph node metastases can be missed on the TMA cores as a result of focal distribution we sampled three TMA cores of each positive lymph node. Eleven primary tumours and seven lymph nodes had no informative results on the TMA because of missing tissue, no tumour tissue or unsuccessful hybridisation. In these cases we analysed a routine tissue sections of the primary tumour by FISH. Whole-tissue section analysis was performed in a further six tumours that were not represented on the TMA. In a further 12 primary tumours, FISH results were already available from previous analyses that had been performed in the diagnostic setting. The characteristics of all 105 patients and their primary tumour are summarised in Table 1. The sites of the metastases are shown in Table 2. The median time between breast cancer diagnosis and the cytological diagnosis of the distant metastases was 66 months (range 0 to 254 months). Eight patients had synchronous metastases (interval less than 1 month). Information on the treatment between the resection of the primary tumour and the occurrence of distant metastases was not available in this study. However, standard adjuvant treatment was performed in accordance with the guidelines of the consensus conference of St Gallen, 2005 [26]. None of the patients with discrepant *HER2 *results received trastuzumab therapy.

**Table 1:** Characteristics of patients and their primary breast cancers

Characteristic	*n *(percentage)
Age (years)	
Mean	57.5
Range	26–85
Tumour size (cm)	
≤ 2	28 (26.6)
>2 ≤ 5	57 (54.3)
>5	9 (8.6)
Not available	11 (10.5)
Histological type	
Ductal	82 (78.1)
Lobular	12 (11.4)
Ductulo-lobular	10 (9.5)
Medullary	1 (1)
Nuclear grade	
1	7 (6.7)
2	41 (39)
3	55 (52.4)
Not available	2 (1.9)

**Table 2:** Localisation of the metastases

Localisation	*n*	Percentage
Ascites	3	2.8
Liver	4	3.8
Lung	9	8.6
Distant lymph nodes	3	2.9
Pericardium	1	1.0
Pleura	74	70.5
Skin/soft tissue	3	2.8
Central nervous system	8	7.6
**Total**	**105**	**100**

### Specimen pretreatment and FISH assay

For FISH analysis of the cytological specimens, the most representative routine Papanicolaou-stained smears or cytospins were used. A hybridisation target area of 22 mm × 22 mm was selected on the basis of high cellularity. This area was first marked with a waterproof pen. In rare tumour cells, the exact locations of the tumour cells in this marked area were saved with relocalisation software (Mark&Find Module; Carl Zeiss Vision GmbH, Halbermoos, Germany) linked to an automated stage (type 00-24-473-0000; Carl Zeiss AG, Oberkochen Germany) on a Zeiss Axioplan 2 epifluorescence microscope (Zeiss, Jena, Germany) before hybridisation. We used this system to relocate the tumour cells after hybridisation in 34 cases (32.4%). The commercially available dual-colour FISH probe PathVysion^® ^was from Abbott/Vysis (Downers Grove, IL, USA). The probes were provided at no charge for those patients in whom FISH analysis was done for research purpose only. It included fluorescence-labelled DNA probes for the *HER2 *gene locus (SpectrumOrange) and centromere 17 (CEP17, SpectrumGreen).

FISH was performed, with small modifications, as recommended by the manufacturer. The smears were washed briefly in xylene until the coverslip could be removed. After that they were washed in fresh xylene twice, 5 minutes per wash. Then they were immersed twice in 100% ethanol for 5 minutes and soaked in 2 × saline sodium citrate (SSC) buffer for 1 minute at room temperature (20–24°C). Subsequently they were incubated in 0.5 mg/ml pepsin solution in 10 mM hydrochloric acid for 10 minutes at 37°C, followed by a wash in phosphate-buffered saline for 5 minutes. The smears were washed twice in Carnoy's fixative for 5 minutes, each time at room temperature, and dehydrated by immersion in 70% ethanol solution for 5 minutes, also at room temperature, followed by immersion in 80% and 100% ethanol. After that the slides were denatured for 10 minutes in 70% formamide/2 × SSC at 73°C. Finally, the smears were dehydrated in a series of 70%, 85% and 100% ethanol (2 minutes per solution), then dried in an oven at 37 to 45°C for 2 minutes.

After denaturation at 73°C for 5 minutes, the probe hybridisation mix was applied to the smears. The smears were then covered with coverslip (22 mm × 22 mm), sealed with rubber cement and incubated overnight in a humid chamber at 37°C. Next morning, the slides were washed in 0.4 × SSC/0.3% Nonidet P40 at 73°C for 2 minutes. Finally, they were rinsed twice in 2 × SSC/0.1% Nonidet P40 for 2 minutes and then air-dried. 4',6-Diamidino-2-phenylindole (DAPI II) was added for counterstaining.

FISH on the histological specimens of the primary tumours was performed as described previously [17]. We analysed the slides with a Zeiss Axioplan 2 epifluorescence microscope (Zeiss, Jena, Germany) equipped with filter sets for DAPI, SpectrumOrange and SpectrumGreen at a magnification of ×1,000. *HER2 *amplification was defined as a *HER2/CEP17 *ratio of 2.0 or more. The number of signals and the *HER2/CEP17 *ratio were first estimated on the tissue specimens of the TMA. The tumours that had an apparently normal ratio and those with an unequivocal amplification (dense clusters of *HER2 *signals or an estimated ratio of more than 2.5) were not analysed again. In the cytological specimens, the ratio was calculated on the basis of 60 scored cells in most cases. In 14 (13%) of the cytological specimens fewer than 60 tumour cells were available for FISH analysis (range 6 to 59 cells).

Cases with a discrepant *HER2 *result between the primary tumour and the metastases were scored again, and at least 20 cells were counted for confirmation. In addition, a routine tissue section of the original donor block was analysed by FISH in discrepant cases in which the primary tumour was represented on the tissue microarray. Thus, a possible sampling bias due to the small size of the TMA cores could be excluded.

### Statistical analysis

The rate of concordance between the primary tumour and metastatic tumour was analysed with the kappa coefficient (κ). A value of κ > 0.8 suggests an 'excellent' concordance in amplification status between the two tumour sites, whereas a κ value between 0.61 and 0.8 indicates 'substantial' agreement. Contingency table analysis was applied to calculate the association between pT category, pN category, nuclear grade and *HER2 *FISH status. Analysis of variance (ANOVA) or Student's *t *test was applied to determine the parameters with greatest influence on the time to metastasis. The level of statistical significance was set at *p *≤ 0.05 two-sided), and all statistical calculations were performed with JMP 3.0 software (SAS Institute Inc., Cary, NC, USA).

## Results

The *HER2 *status of the primary breast cancers and the matched metastases was concordant in 92.4% of the 105 patients at the time of the initial evaluation (κ = 0.76; 95% confidence interval (CI) 0.61 to 0.92). This substantial concordance was increased to 97.1% after re-evaluation of all discordant cases (κ = 0.85; 95% CI = 0.73 to 0.98).

*HER2 *amplification was found in 22 (21%) of the 105 primary tumours and in 21 (20%) of the matched distant metastases. The *HER2 *status between primary tumours and distant metastases differed in eight (7.6%) of the patients at the time of the initial evaluation.

The primary tumours of all eight discrepant cases were reanalysed on routine tissue sections, and the hybridised cytological specimens were carefully scored again. On the routine tissue sections we calculated an exact ratio from at least 20 scored cells. The clinicopathological characteristics and FISH findings of these patients are summarised in Table 3.

**Table 3:** Clinicopathological characteristics and discrepant *HER2 *FISH status between primary tumour and distant metastasis

Case no.	Age (years)	Clinicopathological characteristics				*HER2 *status		
		Tumour size (cm)	Histology	Grade^a^	Metastatic	Metastases	Primary tumour	Explanation^b^
1	34	1.0	Ductal	3	Pleura	Amplified	Negative	True discrepancy
2	52	Not available	Ductal	3	Cerebrospinal fluid	Amplified	Negative	True discrepancy
3	65	2.5	Ductal	3	Pleura	Amplified	Negative	True amplification
4	52	1.6	Ductal	3	Pleura	Negative	Amplified	True amplification
5	47	1.8	Ductal	3	Ascites	Negative	Amplified	Borderline
6	47	Not available	Ductal	3	Pleura	Negative	Amplified	True amplification
7	82	5.2	Ductal	2	Pleura	Negative	Amplified	Borderline
8	62	2.5	Ductal	3	Lung	Negative	Amplified	True negative

*HER2*, gene encoding human EGF-like receptor 2; FISH, fluorescence *in situ *hybridization; true discrepancy, discrepancy remained unexplained; true amplification, amplification was found both in the metastasis and in the primary tumour after review; true negative, amplification was not confirmed after review; borderline, discrepancy was explained by interpretational difficulty due to a *HER2*/CEP17 ratio close to the threshold of 2 or less. ^a^Nuclear grade (1 to 3); ^b^explanations for the discrepancies of *HER2 *status.

**Figure 1:**
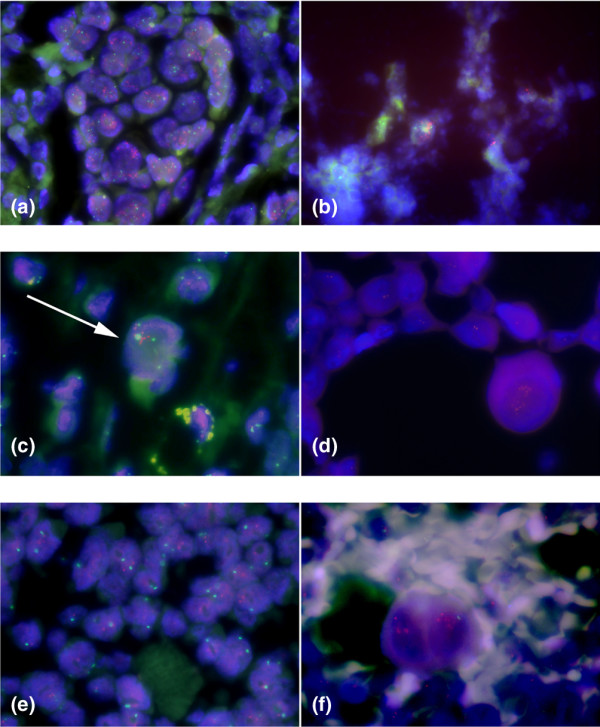
Examples of discrepancies of *HER2 *FISH status between primary breast cancers and matched distant metastases. *HER2 *(gene encoding human EGF-like receptor 2) fluorescence *in situ *hybridisation (FISH) status on histological specimens (a, c, e) and matched cytologies of distant metastases (b, d, f). **(a, b) **Discrepancy due to interpretational difficulty. High-level amplification in primary tumour (a), and rare amplified metastatic cells in pleura effusion that had been overlooked (b). **(c, d) **Discrepancy due to heterogeneity. Rare tumour cells (arrow) with amplification in the primary breast cancer (c), and metastatic cells in pleural effusion with amplification (d). **(e, f) **True discrepancy. *HER2*-negative primary breast cancer (e), and amplification in metastatic cells (f). Original magnifications ×630 to 1,000.

Re-evaluation showed that in one patient with *HER2 *amplification of the primary tumour, rare tumour cells with *HER2 *amplification in the pleural effusion had been overlooked as a result of rarity of malignant cells and a highly predominating background of reactive mesothelial cells and macrophages (Figure 1a,b). In another patient, tumour cells in the pleural effusion but not on the TMA specimen of the primary tumour showed *HER2 *amplification. However, FISH on the routine tissue section of the primary tumour revealed a heterogeneous *HER2 *status with only a few amplified tumour cells (Figure 1c,d).

In another patient, metastasis showed low-level gain but no amplification of the *HER2 *gene (*HER2*/*CEP17 *ratio 1.25, with 3 to 10/4 to 6 signals), whereas the primary tumour had initially been scored as amplified on the TMA spot. Routine section FISH analysis of the primary tumour also revealed low-level gain with a *HER2*/*CEP17 *ratio of 1.6 (with 1 to 2/2 to 6 signals). A further two patients had a 'borderline' amplification. One patient had an average *HER2*/*CEP17 *ratio of 2.1 (with 3 to 9/2 to 4 signals) in the primary tumour but a ratio of only 1.38 in the metastasised cells of the peritoneal effusion (with 3 to 23/3 to 15 signals). The other patient had a *HER2*/*CEP17 *ratio of 2.1, with 2 to 8/2 signals after repeated counting of 20 tumour cells, whereas the metastatic cells had a ratio of 1.69 (5 to 10/3 to 6 signals).

Three patients (2.9%) remained with an unexplained discrepancy even after re-evaluation of the cytological specimens and repeated FISH analysis of the routine tissue sections of the primary tumour (Figure 1e,f). Two of the patients had a primary tumour with a normal copy number of *HER2 *and chromosome 17 but with *HER2 *amplification in the metastasis. The other patient had high-level amplification with a ratio of more than 3 in the primary tumour (10 to 20/2 to 4), in contrast with a ratio of 1.83 (3 to 6/2 to 3) in the metastasis.

*HER2 *amplification was significantly associated with nuclear grade but not with pT and pN category (Table 4). Time from resection of the primary tumour to the diagnosis of metastases was not significantly associated with the pT category (*p *= 0.248), the pN category (*p *= 0.1383) or the nuclear grade (*p *= 0.3777; detailed data not shown). Metastases tended to occur earlier in tumours with *HER2 *amplification than in those without (44.1 ± SEM 7.8 months versus 71.8.6 ± SEM 7.3 months; *p *= 0.0639).

**Table 4:** Comparison between *HER2 *amplified and non-amplified primary breast cancers

Characteristic	*n *amplified (percentage)	*n *not amplified (percentage)	*p*
pT category			
pT1	3 (10.3)	26 (89.7)	0.31
pT2	13 (26.5)	36 (73.5)	
pT3	2 (28.6)	5 (71.4)	
pT4	2 (14.3)	12 (85.7)	
Not available	2 (33.3)	4 (66.7)	
Nodal status			
Positive	14 (21.9)	50 (78.1)	0.44
Negative	4 (14.8)	23 (85.2)	
Not available	4 (28.6)	10 (71.4)	
Histologal type			
Ductal	20 (24.4)	62 (75.6)	0.30
Lobular	2 (16.7)	10 (83.3)	
Ductulo-lobular	0	10 (100)	
Medullary	0	1(100)	
Nuclear grade			
1	0	7 (100)	0.01
2	4 (9.8)	37 (90.2)	
3	18 (32.7)	37 (67.3)	
Not available	0	2 (100)	

*HER2*, gene encoding human EGF-like receptor 2.

The whole metastatic cascade from primary tumour to axillary lymph node metastasis and distant metastasis was analysed in 31 patients (29.5%). One patient with discordant *HER2 *status between the primary tumour (*HER2/CEP17 *ratio 2.1) and the distant metastasis (*HER2/CEP17 *ratio 1.38) had a 'borderline result' in the lymph node (*HER2/CEP17 *ratio 1.9). There was complete concordance of the FISH results in the remaining 30 cases, 4 of which showed amplification.

## Discussion

*HER2 *amplification identifies patients who are likely to respond to therapy with trastuzumab, a humanised antibody directed against the HER2 protein [27,28]. Approval of trastuzumab was originally restricted to patients with *HER2*-positive metastatic disease. On the basis of the positive results of recent international trials [12], approval is now being extended internationally to adjuvant treatment of *HER2*-positive breast cancers. In the mean time, *HER2 *analysis of all newly diagnosed breast cancers has already become a standard in many institutions. Because of the high costs of trastuzumab and the risk of cardiotoxicity in some patients [29], it is crucial to make a precise selection of patients for treatment to guarantee optimal clinical benefit while retaining cost effectiveness.

In patients with metastatic disease, selection for therapy with trastuzumab has traditionally been based on the *HER2 *status of the primary tumour. The reported prevalence of discordance of *HER2 *status between primary tumour and metastasis ranges from 0 to 22.2% when assessment with both immunohistochemistry and FISH is considered [20,21,23,30]. Part of these discrepancies is likely to be due to the well-known technical and interpretational limitations of immunohistochemical *HER2 *assessment [13,15,31]. Because FISH is considered the gold standard for *HER2 *testing [32,33], we investigated the discordance rate based solely on FISH in a large consecutive series of metastatic breast cancers. We found a discrepancy in 8 (7.6%) of the 105 patients. This is in the range of previous studies with FISH and confirms considerable stability of *HER2 *gene status even in tumours that developed distant metastases more than 21 years after initial surgery (Table 5) [19,21]. The discrepancies included three tumours with positive primaries but negative metastases, as determined by FISH, and five positive metastases but negative primaries. There would therefore be a risk of both undertreatment and overtreatment of these metastasised breast cancers if the treatment decision were based only on the *HER2 *status of these primary tumours.

**Table 5:** Fluorescence *in situ *hybridisation studies comparing primary breast cancers and their matched distant metastases

Reference	*n*	Discordance between metastases and primary tumours (percentage)	Number of *HER2*-positive primary tumours	Number of *HER2*-positive metastases
[23]	12	0	12	12
[20]	68	7.0	2	3
[22]	14	0	14	14
[21]	18	22.2	3	7
[19]	17	6.0	Not available	Not available
This study	105	7.6	21	20

*HER2*, gene encoding human EGF-like receptor 2.

The reasons for discrepancy in *HER2 *FISH status between primary breast cancer and metastases have not been investigated in previous studies. Here, detailed re-evaluation of the *HER2 *FISH status by scoring the specimens again or by hybridising routine tissue sections allowed us to discover reasons for *HER2 *discrepancies. Not all discrepant results represented true biological conversion. Instead, we uncovered interpretational difficulties as a reason in five (4.7%) of the patients. One had a slight gain of *HER2 *signals that was regarded as amplified in the first evaluation. In two patients, the discrepancy was explained by a *HER2*/reference ratio that was slightly lower or higher than the threshold of 2, which we refer to as 'borderline'. It is conceivable that such a borderline ratio is more prone to inter-observer variation than amplification with a high ratio or dense gene clusters. In a recent inter-laboratory survey, there was a considerable variability in interpretation of cases with low-level or borderline amplification [34]. In contrast, those authors found excellent reproducibility in *HER2 *FISH analysis for tumours with no amplification or high amplification of the *HER2 *gene. This highlights the need for consensus on the use of an equivocal/borderline interpretative category. Accordingly, the package insert of the PathVysion includes the statement 'a ratio at or near the cutoff (1.8 to 2.2) should be interpreted with caution'. The clinical relevance of this type of interpretational limitation is not clear, because the impact of the ratio level on the likelihood of therapy response is still unknown. One could hypothesise that the response rate is low in breast cancers with a borderline FISH result.

Rarity of *HER2 *amplified cells was responsible for a discrepancy in a further two patients. In one of these, rare *HER2*-amplified cells were initially overlooked on a cytological smear from metastasis with a high background of reactive cells. In the other patient, rare amplified cells were not detected initially because of intratumoral heterogeneity of *HER2 *status within the primary tumour. In this patient, we must assume the presence of clonal selection of the rare *HER2*-amplified cells in the primary for distant metastatic spread. Our finding also emphasises the importance of careful and thorough evaluation of the hybridised specimens, because heterogeneity with small cancer foci prevails in rare cases [35,36].

In three of the eight patients (patients 1, 2, and 8 in Table 3) we could not identify any interpretational reason for the discrepancy. Two of these were negative, as determined by FISH, in the primary but positive in the metastasis. In the third tumour, high-level amplification in the primary contrasted with low-level gain in the metastasis. It is possible that these three tumours represented true conversion of *HER2 *status by clonal selection or genetic drift during metastatic progression. However, we cannot exclude the possibility of undetected heterogeneity even in these cases, because only a small percentage of the entire tumour volume is represented on a histological section or on a cytological smear [37,38].

The patients of this study were selected on the basis of the availability of cytologically diagnosed distant metastases. Fine-needle aspiration cytology of solid lesions or exfoliative cytology (for example malignant effusions) is a commonly used method to diagnose or confirm metastatic disease. Our results confirm that cytological specimens are well suited to *HER2 *FISH analysis [22,39,40]. It has previously been shown that results of *HER2 *FISH analyses from cytological specimens are highly concordant with matched histological sections [41,42]. It is therefore unlikely that our results were affected by the different types of tumour material of primary tumours and metastases.

The standard morphological parameters (pT category, pN category and grade) are strong prognostic factors in newly diagnosed breast cancer [43]. Interestingly, none of these parameters was significantly associated with the time interval from initial treatment to cytological diagnosis of distant metastasis in our selected series of patients. Because the time of cytological sampling of metastatic cells is not always identical with the time of first clinical detection of metastasis, prognostic data must be interpreted with caution in our study. Nevertheless, our data indicate that within the group of breast cancers that are capable of distant metastasis, the dynamics of metastasis is driven by biological or environmental factors that are not fully reflected by morphological features. The observed tendency of *HER2 *amplification towards early metastasis is concordant with the known adverse prognostic role of *HER2 *amplification [44].

## Conclusion

*HER2 *FISH status is highly preserved as breast cancers progress to metastatic disease. However, a discrepancy in *HER2 *status exists in a small fraction of patients. As well as true conversion of *HER2 *status, interpretational difficulties due to borderline FISH results, rarity of tumour cells and intratumoral heterogeneity were identified as important reasons for the discrepancy. Irrespective of the reason, however, every discordance in *HER2 *status is a diagnostic reality and can lead to inaccurate treatment decisions with medical and economical effects. To guarantee optimal care of individual patients, we advocate *HER2 *analysis of distant metastases in all patients irrespective of the result in the primary tumour. The feasibility of this approach will ultimately be determined by economic factors. Future patients with distant metastases after adjuvant treatment for *HER2*-positive breast cancer will pose additional diagnostic and therapeutic challenges. It is possible that treatment with trastuzumab leads to clonal selection of *HER2*-negative tumour cells, as observed in individual patients [35]. Thus, *HER2 *testing of metastasis may become a necessity in every patient.

## Abbreviations

*CEP17 *= centromere probe of chromosome 17; CI = confidence interval; DAPI = 4',6-diamidino-2-phenylindole; FISH = fluorescence *in situ *hybridisation; *HER2 *= gene encoding human EGF-like receptor 2; SSC = saline sodium citrate; TMA = tissue microarray.

## Competing interests

LB has received speaker's honorarium and refunding of travel costs, and research funding from Abbott Molecular Inc. CT has received refunding of travel costs by Abbott Molecular Inc. All other authors declare that they do not have competing interests.

## Authors' contributions

CT contributed to the study design, was responsible for statistical evaluation and drafted the manuscript. SS participated in study design and FISH evaluation. UW, RS and GC selected and provided matched specimens. HN performed and scored FISH on the histological specimens. BG, MH and BDV performed and scored FISH on the cytological specimens. IZ performed statistical analyses. LT was responsible for and supervised FISH on the histological specimens. GF was involved in evaluation of the cytological specimens. LB conceived the study, supervised the experiments, revised the manuscript critically for important intellectual content and gave final approval of the version to be published. All authors read and approved the final manuscript.
